# Identification of cyst nematode B-type CLE peptides and modulation of the vascular stem cell pathway for feeding cell formation

**DOI:** 10.1371/journal.ppat.1006142

**Published:** 2017-02-03

**Authors:** Xiaoli Guo, Jianying Wang, Michael Gardner, Hiroo Fukuda, Yuki Kondo, J. Peter Etchells, Xiaohong Wang, Melissa Goellner Mitchum

**Affiliations:** 1 Division of Plant Sciences and Bond Life Sciences Center, University of Missouri, Columbia, Missouri, United States of America; 2 College of Plant Science and Technology, Huazhong Agricultural University, Wuhan, China; 3 Department of Biological Sciences, Graduate School of Science, University of Tokyo, Bunkyo-ku, Tokyo, Japan; 4 Department of Biological Sciences, Durham University, Durham, United Kingdom; 5 School of Integrative Plant Science, Cornell University and Robert W. Holley Center for Agriculture and Health, United States Department of Agriculture, Agricultural Research Service, Ithaca, New York, United States of America; University of California, Riverside, UNITED STATES

## Abstract

Stem cell pools in the SAM (shoot apical meristem), RAM (root apical meristem) and vascular procambium/cambium are regulated by CLE-receptor kinase-WOX signaling modules. Previous data showed that cyst nematode CLE-like effector proteins delivered into host cells through a stylet, act as ligand mimics of plant A-type CLE peptides and are pivotal for successful parasitism. Here we report the identification of a new class of CLE peptides from cyst nematodes with functional similarity to the B-type CLE peptide TDIF (tracheary element differentiation inhibitory factor) encoded by the *CLE41* and *CLE44* genes in Arabidopsis. We further demonstrate that the TDIF-TDR (TDIF receptor)-WOX4 pathway, which promotes procambial meristem cell proliferation, is involved in beet cyst nematode *Heterodera schachtii* parasitism. We observed activation of the TDIF pathway in developing feeding sites, reduced nematode infection in *cle41* and *tdr-1 wox4-1* mutants, and compromised syncytium size in *cle41*, *tdr-1*, *wox4-1* and *tdr-1 wox4-1* mutants. By qRT-PCR and promoter:*GUS* analyses, we showed that the expression of *WOX4* is decreased in a *clv1-101 clv2-101 rpk2-5* mutant, suggesting that WOX4 is a potential downstream target of nematode CLEs. Exogenous treatment with both nematode A-type and B-type CLE peptides induced massive cell proliferation in wild type roots, suggesting that the two types of CLEs may regulate cell proliferation during feeding site formation. These findings highlight an important role of the procambial cell proliferation pathway in cyst nematode feeding site formation.

## Author summary

Cyst nematodes are one of the most economically important groups of plant-parasitic nematodes worldwide. These parasites not only damage root systems during infection, but their obligate sedentary endoparasitic lifestyle requires them to induce the formation of a unique feeding cell within host roots where they drain life-sustaining nutrients that ultimately lead to plant disease symptoms such as stunting, chlorosis, wilting, and yield loss. The formation of a feeding cell by the nematode requires manipulation of host developmental pathways. For this, the nematode secretes peptides into host cells that function as mimics of plant peptides to commandeer host developmental programs for their own benefit. Currently, the downstream signaling pathways activated to initiate developmental cascades that are required for cellular programming into a feeding cell are not known. Here, we discovered a new class of plant peptide mimics secreted by cyst nematodes and identified the transcription factor WOX4 as a downstream target of nematode peptide signaling to reveal for the first time an important role for the vascular stem cell pathway in feeding cell formation. Understanding the molecular basis of plant-nematode interactions can lead to the development of novel strategies to control these major agricultural pests.

## Introduction

Cyst nematodes feed as obligate sedentary endoparasites by successfully exploiting cells near the root vasculature to obtain plant nutrients. Infective juveniles select a single cell, typically a procambial or pericycle cell, to initiate the formation of a feeding site called a syncytium. The syncytium is formed by cell division and progressive cell wall dissolution surrounding the initial feeding cell followed by the fusion of hundreds of cells, resulting in a permanent feeding structure which provides nutrients to support nematode growth and development [1]. To orchestrate feeding cell formation, the nematode uses its stylet (hollow mouth spear) to deliver a suite of effector proteins into the selected cell, including CLAVATA3/EMBRYO SURROUNDING REGION (CLE)-like peptides [2–4]. Nematode CLEs remain the only CLE peptides identified outside the plant kingdom [5, 6].

Plant CLEs are small secreted peptides involved in the maintenance of stem cell pools and the precise determination of cell fate [7–9]. In Arabidopsis, there are 32 CLE members corresponding to 26 CLE peptides that are classified as either A-type or B-type CLE peptides. The A-type CLE peptides including CLV3 suppress shoot and root apical meristem activity and promote cell differentiation, whereas the B-type peptides (CLE41-CLE44) suppress differentiation of tracheary elements and promote procambial cell division. Since the expression of many *CLE* genes overlaps, potential antagonistic or synergistic interactions exist among CLEs. It has been shown that CLE6 potentiates the activity of CLE41 to induce vascular cell proliferation and delay differentiation, providing an example of synergism between A and B-type CLE peptides in a cell-specific context [10]. To date, only nematode CLE genes encoding plant A-type CLE mimics have been cloned from different cyst nematodes and shown to be required for successful parasitism [6, 11–16].

In Arabidopsis, CLE-receptor kinase-WOX signaling modules are conserved in meristematic stem cell maintenance in the shoot apical meristem (SAM), root apical meristem (RAM), and vascular meristem (procambium/cambium) [8]. In the SAM, a homeodomain transcription factor WUSCHEL (WUS), which resides in the organizing center (OC) of the SAM, promotes stem cell proliferation. WUS binds to the promoter of *CLV3* to activate its expression in a negative feedback loop. The CLV3 signal is perceived by receptor complexes including CLAVATA1 (CLV1), the CLAVATA2 (CLV2)/CORYNE (CRN) heterodimer and RECEPTOR-LIKE PROTEIN KINASE 2 (RPK2), and transmitted via a downstream pathway that leads to the repression of *WUS* expression [17–23]. In the RAM, *WUSCHEL-RELATED HOMEOBOX5* (*WOX5*) expressed in the quiescent center (QC) is required to maintain the distal stem cell pool. The CLE40 signal is perceived by ARABIDOPSIS CRINKLY4 (ACR4) and CLV1 receptors to inhibit *WOX5* expression and then promote stem cell differentiation [24–26].

The B-type CLE peptides function in the vascular meristem which consists of procambial cells. Tracheary Element Differentiation Inhibitory Factor (TDIF), encoded by *CLE41* and *CLE44*, regulate two developmental processes including the division of procambial cells and the differentiation of procambial cells into xylem [27–31]. TDIF RECEPTOR/PHLOEM INTERCALATED WITH XYLEM (TDR/PXY) perceives TDIF and activates two separate downstream pathways for the regulation of these two processes. A transcription factor WOX4 is targeted to promote procambial cell proliferation, whereas Glycogen Synthase Kinase 3 proteins (GSK3s) which are activated via physical association with TDR, are involved in the suppression of xylem differentiation. A transcription factor BRI1-EMS SUPPRESSOR 1 (BES1) is suppressed by the TDIF-TDR-GSK3 pathway which in turn inhibits xylem differentiation [32, 33]. The TDIF-TDR-WOX4 pathway and TDIF-TDR-GSK3-BES1 pathway act independently in procambial cells to govern vascular stem cell balance.

Given the important role of CLE signaling in plant growth and development, it is not surprising that cyst nematodes would develop peptide mimics to tap into important developmental pathways and reprogram them for feeding site formation. Recently, we showed that CLE receptor CLV1, the CLV2/ CRN receptor complex and RPK2, which transmit the CLV3 signal in the SAM, are involved in nematode CLE peptide perception and proper feeding cell formation in Arabidopsis and soybean [34–36]. However, the downstream signaling pathways activated to initiate developmental cascades that are required for cellular reprogramming into a syncytium are currently unclear. In this paper, we identified B-type CLE mimics from cyst nematodes and investigated the role of these peptides in nematode parasitism. Significantly, we showed that WOX4 is a potential downstream target of nematode CLE signaling, highlighting the importance of TDIF-TDR-WOX4 mediated procambial cell proliferation for feeding site formation.

## Results

### B-type CLE mimics were identified from cyst nematodes

**Fig 1:**
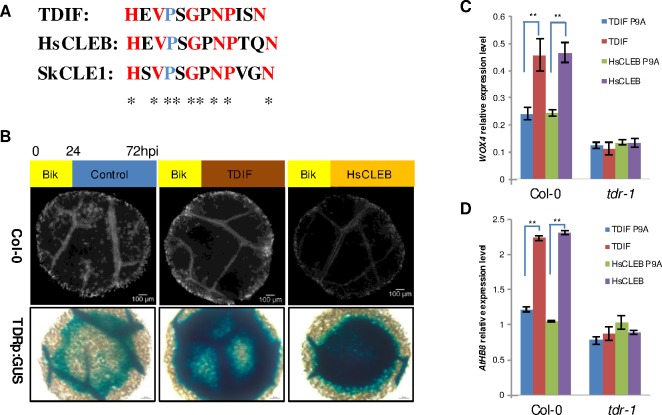
Identification of B-type CLE mimics from cyst nematodes. (A) 12-aa alignment of nematode B-type CLE peptide with Arabidopsis TDIF and *Selaginella kraussiana* TDIF (SkCLE1) (B) Suppression effects of Arabidopsis TDIF and HsCLEB on tracheary element differentiation. Leaf disks from wild-type and TDRp:GUS plants were precultured with bikinin and then were transferred to media without bikinin or with Arabidopsis TDIF or HsCLEB. (C) Relative expression level of *WOX4* and *AtHB8* in Col-0 and *tdr-1* seedlings that were treated with Arabidopsis TDIF P9A, Arabidopsis TDIF, HsCLEB P9A, and HsCLEB for 7 days. *AtUBC21* gene (At5g25760) was used as internal control. Error bars represent SD of the means (n = 3). Asterisks indicate statistically significant differences according to Student’s *t*-test (*P* < 0.01). The experiments were repeated three times with similar results.

A-type CLE mimics were previously identified from gland-enriched cDNA libraries of soybean cyst nematode (*Heterodera glycines*) [12, 13, 37, 38] and subsequently cloned from other cyst nematode species [15, 39, 40], but whether these nematodes also have B-type CLE mimics has remained elusive. To determine whether cyst nematodes secrete B-type CLE mimics, we used the TDIF sequence from Arabidopsis to identify orthologous sequences in a recently annotated soybean cyst nematode transcriptome generated from early parasitic life stages by coupling deep sequencing with *de novo* assembly. We identified three transcripts encoding B-type CLEs, suggesting that these CLEs belong to a small gene family in the nematode. Here we refer to these three transcripts as c21342_g1_i1, c21342_g1_i2, and c21342_g1_i5 (S1A Fig). c21342_g1_i1 and c21342_g1_i5 shared identical open reading frame sequences, encoding a 105 amino acid (aa) protein with a predicted 23 aa signal peptide at the N-terminus for secretion. c21342_g1_i2 was identical to c21342_g1_i1 and c21342_g1_i5 except that it harbored a unique stretch of 35 aa immediately upstream of the C-terminal CLE domain (S1B Fig). The c21342_g1_i2 transcript may harbor a retained intron, which represents a form of alternative splicing commonly observed in nematode effector sequences [41]. Primers designed based on the UTR region sequences of SCN B-type *CLEs* were used to confirm expression by cloning the corresponding cDNA by RT-PCR from RNA extracted from parasitic J2s. One cDNA sequence, named *HgCLEB1*, was cloned and sequenced. The same primers were used to identify orthologous sequences encoding B-type CLEs from the beet cyst nematode *H*. *schachtii*. Two cDNA sequences, named *HsCLEB1* and *HsCLEB2*, were cloned by RT-PCR from RNA extracted from parasitic J2s. The sequences were confirmed from multiple independent PCR reactions. All three sequences encoded secreted proteins. Interestingly, the 12-aa B-type CLE peptide sequence (HEVPSGPNPTQN) was not only identical within a species but across species (S1C Fig). A-type *CLE* genes are highly upregulated specifically within the dorsal gland cell of parasitic life stages of cyst nematodes [13, 14, 16, 42]. However, our *in situ* hybridization attempts to determine if the same localization pattern was true for B-type *CLEs* were unsuccessful. A comparison of the relative expression levels of A- and B-type *CLEs* in the *H*. *glycines* transcriptome indicated that the significantly lower expression of B-type *CLEs* compared to A-type CLE *HgCLE2* (S1D Fig) likely precluded us from successfully detecting B-type *CLEs* in gland cells. Sequence alignment revealed a high level of sequence conservation between the nematode and vascular plant TDIF peptides. We found 2 aa substitutions between HsCLEB and Arabidopsis TDIF and 3 aa substitutions between HsCLEB and spikemoss *Selaginella kraussiana* SkCLE1 [43] (Fig 1A). Those substitutions were reported to not be essential for the activity of TDIF during xylem differentiation [31].

TDIF plays a crucial role in procambial cell maintenance by suppressing their differentiation into xylem cells and promoting their proliferation in Arabidopsis [32]. In order to test whether the nematode CLE peptide had a similar function as TDIF in suppressing xylem cell differentiation, an *in vitro* leaf disc culture system was employed [44]. It has been reported that GSK3s function to inhibit xylem differentiation downstream of TDIF-TDR signaling and repression of GSK3s activity with bikinin can induce xylem differentiation [33]. Therefore, we examined the effect of TDIF and nematode peptide on xylem differentiation after preculturing leaf discs with bikinin for 24 h. Treatment of precultured leaf disks with TDIF or nematode peptide for 48 h severely suppressed xylem differentiation and caused a high level of *TDRp*:*GUS* expression. Our results indicate that nematode peptide can suppress xylem differentiation similar to TDIF (Fig 1B). Furthermore, TDIF regulates procambial stem cell proliferation via WOX4. qRT-PCR analysis showed that the expression of *WOX4* and another procambium marker gene *AtHB8* were induced by TDIF treatment as well as nematode HsCLEB peptide treatment in 7-day-old wild type seedlings compared with their respective nonfunctional peptide (TDIF P9A and HsCLEB P9A), whereas this induction was not observed in TDIF receptor mutant *tdr-1* (Fig 1C and 1D). Together, these data suggest that this nematode peptide acts as a ligand mimic of the plant B-type TDIF peptide.

### TDIF-TDR-WOX4 pathway is responsive to nematode infection

**Fig 2:**
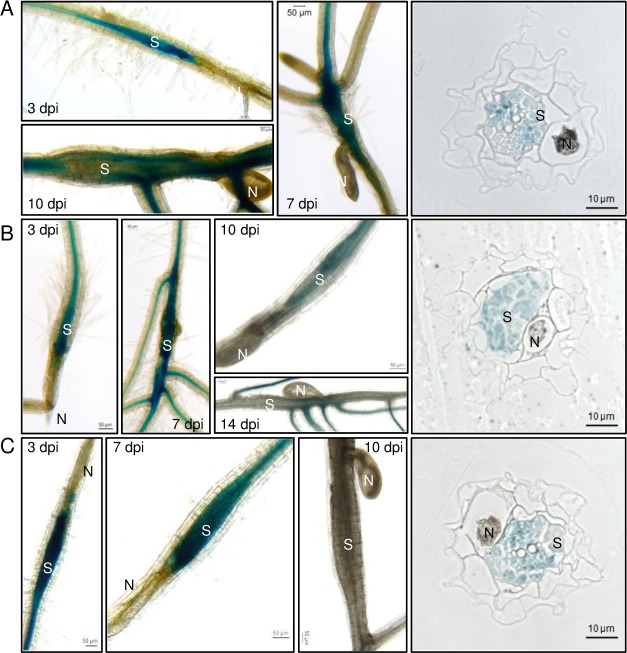
Expression pattern of TDIF-TDR-WOX4 pathway upon nematode infection. Three Arabidopsis promoter:GUS lines including (A) TDRp:GUS. (B) WOX4p:GUS. (C) CLE41p:GUS were infected with *H*. *schachtii*. GUS staining was performed at different time points (days) after inoculation (dpi). For cross-sectioning, roots at 3 dpi were used. N = nematode; S = syncytium.

Next we investigated the involvement of B-type CLE signaling in nematode infection. CLE41p:GUS, TDRp:GUS and WOX4p:GUS transgenic Arabidopsis lines were infected with BCN and examined during nematode development to see if nematode infection could induce the expression of these genes at the feeding site. In non-infected roots, GUS expression was restricted to the vasculature, especially at the lateral root junction, as described previously [29, 30] (S2 Fig). In addition, all three lines demonstrated expression of GUS specifically within developing syncytia during the early stages of nematode infection including J2 (3 days post-inoculation; dpi) and J3 (7 dpi) stages. GUS expression was undetectable within syncytia of TDRp:GUS and CLE41p:GUS lines by 10 dpi. At 10 dpi, expression was reduced within syncytia of WOX4p:GUS lines and undetectable by the time nematodes reached the J4 stage (14 dpi) (Fig 2). As a comparison, the expression of *WUS* and *WOX5* was absent in the developing syncytium although *WOX5* expression was observed in the apical meristem of lateral roots originating from the nematode feeding site (S2 Fig).

### *TDR* is expressed in the initial syncytial cell and adjacent cells

**Fig 3:**
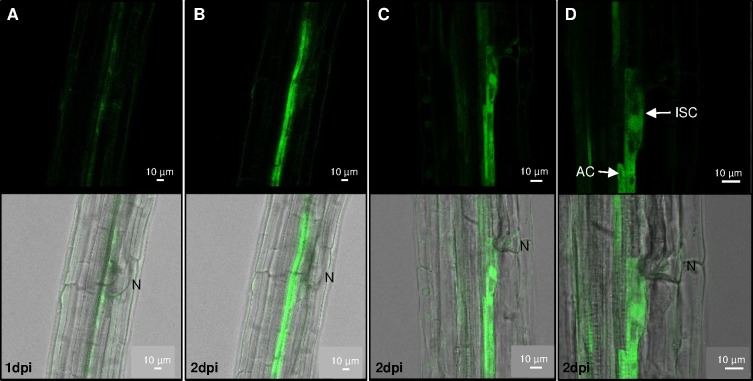
Expression of *TDR* at early stages of nematode infection. TDRp:GFP expression was evaluated at nematode feeding sites after 1 dpi (A) and 2 dpi (B-D). (A) and (B) pictures the same nematode feeding site. (D) is a higher magnification of the feeding site shown in (C). N = nematode; AC = adjacent cell; ISC = initial syncytial cell.

Syncytium formation requires progressive cell wall dissolution of the initial syncytial cell (ISC) followed by the fusion of adjacent cells (AC). In order to better resolve the temporal dynamics of nematode CLE signaling during early stages of syncytium formation, a TDRp:GFP transgenic Arabidopsis line was utilized to evaluate the precise expression of *TDR* during syncytium formation. By following the same infection site, TDRp:GFP expression was monitored at 1 dpi and 2 dpi. GFP was upregulated in root cells associated with the nematode feeding site. Upon nematode infection, *TDR* expression was found both within the ISC and AC (Fig 3A–3D). These data indicate that *TDR* is expressed in the syncytium and in cells to be incorporated into the syncytium.

### TDR signaling is important for syncytium formation

**Fig 4:**
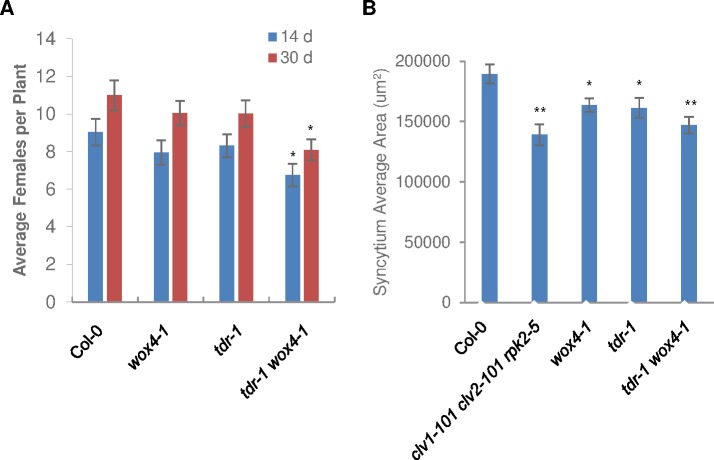
Involvement of TDIF-TDR-WOX4 in syncytium formation. (A) Nematode infection is decreased in *tdr-1 wox4-1* mutant. Average number of females per plant was counted at 14 d and 30 d post inoculation. (B) Syncytium size is reduced in *tdr-1*, *wox4-1*, and *tdr-1 wox4-1* mutants. Syncytium area was measured at 14 d post inoculation. Error bars represent SE of the means (n = 36). Asterisks indicate statistically significant differences compared with Col-0 by Student’s *t*-test (*P < 0.05 and **P < 0.01). The experiments were performed three times with similar results.

To determine if the TDR signaling pathway is involved in nematode infection, we performed nematode infection assays on the corresponding Arabidopsis mutant lines *tdr-1*, *wox4-1*, and *tdr-1 wox4-1*. We did not observe a significant reduction in infection on the single mutants, except for *pxy-3* at 30 dpi, which is another allele of *TDR* (S3 Fig). Both *tdr-1* and *pxy-3* are null alleles, but it is unknown whether *pxy-3* is a stronger mutant allele than *tdr-1*. We did observe a significant reduction in nematode infection on the *tdr-1 wox4-1* mutant (Fig 4A). Similar results were obtained with other mutant allele combinations including *pxy-3*, including *pxy-3 wox4-1*, *pxy-3 wox14-1*, and *pxy-3 wox4-1 wox14-1* (S3 Fig). To further investigate whether the WOX4-mediated cell proliferation pathway is crucial for feeding site formation, we examined syncytium size in the mutants. We observed a significant reduction in syncytium size in *tdr-1*, *wox4-1*, and *tdr-1 wox4-1* mutants relative to that of wild type (Fig 4B). These results indicate that the TDR signaling pathway plays an important role in feeding site formation.

### WOX4 is a potential downstream target of nematode A-type CLE signaling

Previous results have demonstrated that CLV1, CLV2, and RPK2 which transmit the CLV3 signal for shoot apical meristem maintenance are important for nematode parasitism [34–36]. *clv1-101 clv2-101 rpk2-2* showed a 50–60% reduction in nematode infection and reduced syncytium size. However, this mutant is male sterile which presented a challenge for further functional characterization. Therefore, we generated a new mutant allele *clv1-101 clv2-101 rpk2-5* which is partially sterile. As expected, the new triple mutant shows shoot phenotypes similar to the *clv3* mutant with enlarged shoot and floral meristems due to uncontrolled proliferation of stem cells. The triple mutant also has longer roots compared with wild type (S4 Fig). Consistent with the same mutant combination containing *rpk2-2*, *clv1-101 clv2-101 rpk2-5* was highly resistant to nematode infection and syncytium size was compromised (S4 Fig and Fig 4B). We then used the *clv1-101 clv2-101 rpk2-5* mutant, defective in A-type CLE perception, to identify downstream signaling components that are regulated by these receptors.

**Fig 5:**
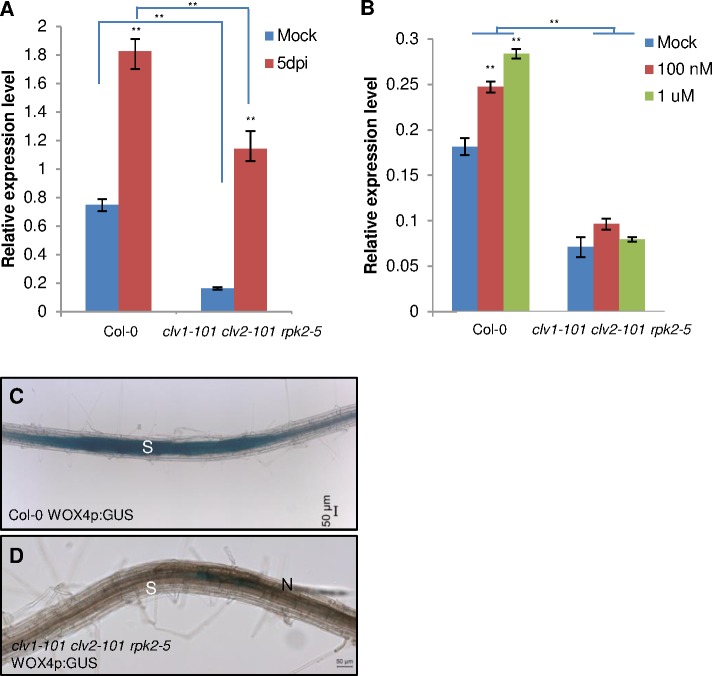
Reduced *WOX4* gene expression in *clv1-101clv2-101rpk2-5* mutant roots. (A) *WOX4* gene expression under nematode infection. Small root pieces containing nematode infection sites were cut from wild type and *clv1-101 clv2-101 rpk2-5* mutant plants at 5 d after nematode inoculation. (B) *WOX4* gene expression under HsCLE2 peptide treatment. Wild type and *clv1-101 clv2-101 rpk2-5* plants were grown on vertical plates without and with HsCLE2 peptide for 5 days and whole roots were cut for qRT-PCR analysis. For qRT-PCR, *AtUBC21* gene (At5g25760) was used as internal control. Error bars represent SD of the means (n = 3). Asterisks indicate statistically significant differences using Student’s *t*-test (**P < 0.01). The experiments were carried out three times with similar results. (C)-(D) *WOX4p*:*GUS* expression at nematode feeding sites in wild type and *clv1-101 clv2-101 rpk2-5* backgrounds. GUS staining was performed at 3 d post inoculation. N = nematode; S = syncytium.

First, qRT-PCR revealed that the expression of *WOX4* was decreased in the *clv1-101 clv2-101 rpk2-5* mutant. Although nematode infection can induce *WOX4* expression, the expression in the triple mutant was still lower compared with the expression in wild type (Fig 5A). Similar results were obtained with exogenous treatment of roots with *H*. *schachtii* A-type CLE peptide HsCLE2 (identical to AtCLE5/6). HsCLE2 peptide treatment upregulated the expression of *WOX4* in wild type roots; however, the upregulation was barely seen in the *clv1-101 clv2-101 rpk2-5* mutant (Fig 5B). In order to confirm this, WOX4p:GUS was crossed into *clv1-101 clv2-101 rpk2-5*. We then examined GUS expression in nematode feeding sites. Strong GUS expression was detected in nematode feeding sites of the wild type (Fig 5C), whereas we observed decreased GUS expression in the *clv1-101 clv2-101 rpk2-5* mutant (Fig 5D). This suggested that *WOX4* is a potential downstream target of nematode CLE signaling dependent on the receptors CLV1, CLV2, and RPK2.

### Expression of *CLE41* is CLV1 CLV2 RPK2 dependent and required for syncytium formation

**Fig 6:**
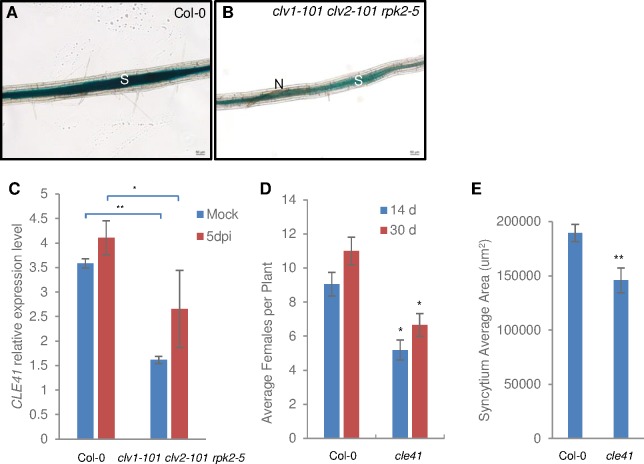
Decreased *CLE41* gene expression in *clv1-101 clv2-101 rpk*2-5 mutant and reduced nematode development on the *cle41* mutant. (A)-(B) *CLE41p*:*GUS* expression in nematode feeding sites of wild type and *clv1-101 clv2-101 rpk*2-5 background. (C) qRT-PCR analysis of *CLE41* gene expression in wild type and *clv1-101 clv2-101 rpk*2-5 mutant at 5 dpi. Nematode development (D) and syncytium size (E) is reduced in the *cle41* mutant. Error bars represent SD of the means (n = 3). Asterisks indicate statistically significant differences by Student’s *t*-test (*P < 0.05 and **P < 0.01). The experiments were repeated three times with similar results.

Since *WOX4* is a downstream target of CLE41, we then tested the expression of *CLE41* in the *clv1-101 clv2-101 rpk2-5* triple mutant background in order to identify the components that possibly mediate *WOX4* regulation. CLE41p:GUS was crossed into the *clv1-101 clv2-101 rpk2-5* mutant. GUS staining of nematode feeding sites showed that the expression of *CLE41* is decreased in the *clv1-101 clv2-101 rpk2-5* mutant line (Fig 6A and 6B), suggesting that downregulation of *WOX4* expression in the *clv1-101 clv2-101 rpk2-5* mutant is at least partially due to the decreased expression of *CLE41*. Further qRT-PCR analysis showed a consistent expression pattern of *CLE41* in nematode infection sites (Fig 6C). Nematode development and syncytium size were compromised on the *cle41* mutant indicating that both endogenous CLE41 and nematode-derived B-type CLEs are required for proper establishment of feeding cells (Fig 6D and 6E).

### Nematode A- and B-type CLE peptides regulate proliferation of vascular cells

The above data showed that nematodes may secrete both A-type and B-type CLE peptides into root cells and *WOX4* acts as a potential downstream target of nematode CLE peptides, so we tested the direct effect of CLE peptides on the expression of *WOX4*. Exogenous treatment with both A-type and B-type peptide induce the expression of *WOX4* in the root, but *WOX4* expression was lower in *tdr-1 clv1-101 clv2-101* mutant subjected to the treatment (S5 Fig). Through cross-sectioning of the root tissue, we found that the diameter of the *tdr-1 clv1-101 clv2-101* mutant is smaller relative to that of the wild type with or without nematode infection (S6A–S6G Fig). The *tdr-1 clv1-101 clv2-101* mutant is highly resistant to nematode infection similar to *clv1-101 clv2-101 rpk2-5* mutant (S6H Fig). In the aboveground, the rosette leaves of the *tdr-1 clv1-101 clv2-101* mutant were narrower compared with that of the *clv1-101 clv2-101* mutant (S6I–S6K Fig).

**Fig 7:**
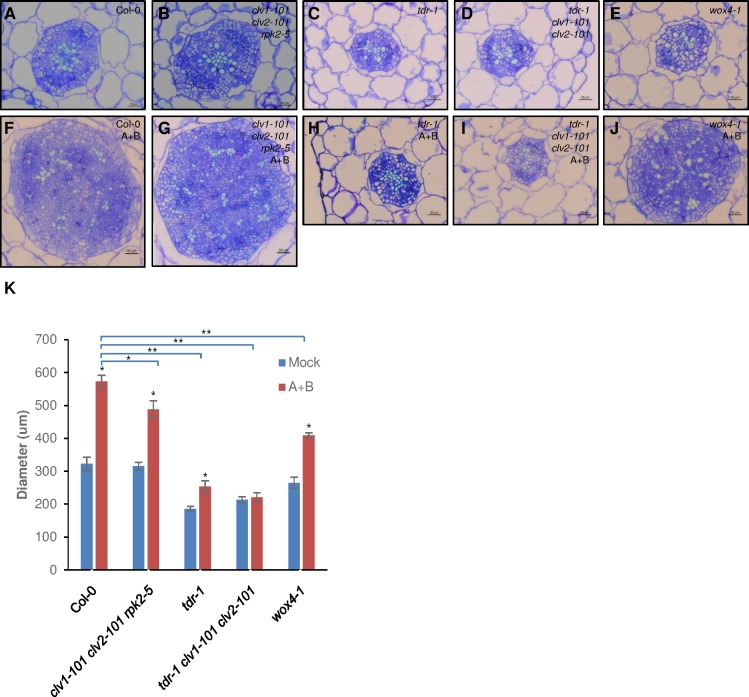
Combined effect of nematode HsCLE2 and HsCLEB peptides on vascular cell proliferation. Seedlings including Col-0, *clv1-101 clv2-101 rpk2-5*, *tdr-1*, *tdr-1 clv1-101 clv2-101*, and *wox4-1* were grown for 10 d in liquid medium supplemented without (A-E) or with HsCLE2 and HsCLEB peptides (5 μM each) (F-J). Hypocotyls were sectioned and stained with toluidine blue. (K) Vascular diameter measurement of hypocotyl sections (n = 5 to 8). Asterisk indicates *P* < 0.05 (*) or P < 0.01 (**) by using Student’s *t*-test.

It has been reported that A-type AtCLE5/6 peptide can potentiate the activity of B-type AtCLE41 peptide to induce vascular cell proliferation in hypocotyl in exogenous peptide assays [10]. Cross sectioning of the hypocotyl was performed after exogenous treatment with the corresponding A- and B-type peptides from the nematode, and we observed similar results. Massive cell proliferation was observed in peptide-treated Col-0 and *clv1-101 clv2-101 rpk2-5* triple mutant (Fig 7A, 7B, 7F and 7G). The *wox4-1* mutant showed less cell proliferation compared with wild type after peptide treatment (Fig 7E and 7J). However, cell proliferation was significantly reduced in peptide-treated *tdr-1* and *tdr-1 clv1-101 clv2-101* mutants (Fig 7C, 7D, 7H and 7I). Diameter measurement data showed that although peptide treatment can promote massive cell division in the *clv1-101 clv2-101 rpk2-5* and *wox4-1* mutants, the extent of the induction is lower compared with wild type (Fig 7K). Only a small effect of the treatment was observed on the *tdr-1* mutant compared with wild type, but no effect was observed on *tdr-1 clv1-101 clv2-101* mutant, indicating that TDR is pivotal for A/B peptide-induced cell proliferation.

## Discussion

Nematode *CLE* genes have been cloned from different cyst nematodes including soybean cyst nematode *H*. *glycines* [9, 14, 41, 42], potato cyst nematode *Globodera rostochiensis* [39], beet cyst nematode *H*. *schachtii* [15, 42], tobacco cyst nematode *G*. *tabacum* [40] and more recently from the reniform nematode *Rotylenchulus reniformis* [45]. Like cyst nematodes, the reniform nematode also induces the formation of a syncytium. The nematode *CLE* genes are expressed in the dorsal gland of parasitic life stages and cyst nematode CLEs have been demonstrated to be required for successful nematode parasitism [13, 14, 39, 42, 46]. Overexpression of cyst nematode *CLE* genes and exogenous peptide treatment demonstrated that nematode CLEs identified to date act as ligand mimics of plant A-type CLEs [14, 15]. Our prior studies have shown that plant CLE receptors including CLV1, CLV2/CRN, and RPK2 receptor complexes from Arabidopsis and soybean are required for nematode infection and proper feeding site formation, suggesting host plant CLE receptors can perceive nematode CLE signals [10, 35, 36]. Nevertheless, the exact function of nematode CLEs for feeding site formation remains elusive. In this study, we report on the discovery of B-type CLE TDIF mimics from cyst nematodes and illustrate the importance of the TDIF-mediated cell proliferation pathway in nematode feeding site formation.

Plant CLEs have been classified into A-type and B-type CLE peptides based on their ability to promote or suppress stem cell proliferation or differentiation. Previous data have shown that nematode CLEs are able to mimic A-type CLE function *in planta*, and might be used to manipulate the host plant CLE signaling. Feeding site formation is a complicated process that is controlled in time and space by the secretion of nematode effectors [6]. It has been hypothesized that simultaneous secretion of a mixture of different CLE peptides from nematodes into host cells aids in the initiation and maintenance of feeding sites. This potential led us to search for B-type CLE peptides secreted from the nematode during parasitism. Recent deep sequencing of parasitic stages of the soybean cyst nematode facilitated a *de novo* assembly of the transcriptome and a bioinformatics approach to identify new CLE effectors secreted by the nematode. As a result, multiple *CLE* genes were isolated from both soybean and beet cyst nematodes. Interestingly, the corresponding 12-aa peptide (HEVPSGPNPTQN) encoded by the new *CLE* genes is identical within and across species. The peptide sequence shows high similarity with plant B-type CLE TDIF, with only 2 aa substitutions between HsCLEB and Arabidopsis TDIF and 3 aa substitutions with SkCLE1 [43].

The involvement of TDIF signaling in vascular meristem maintenance is the most well-characterized B-type CLE signaling pathway [32]. In contrast to A-type CLE peptides, TDIF does not inhibit shoot or root apical meristem development. The TDIF dodecapeptide isolated from a *Zinnia* xylogenic culture system specifically suppressed tracheary element differentiation while promoting cell division [31]. The TDIF peptide is encoded by *CLE41* and *CLE44* in Arabidopsis. Exogenous TDIF treatment not only promotes the proliferation of procambium, but also inhibits their differentiation into xylem in Arabidopsis. Genetic and biochemical evidence revealed that the TDIF receptor TDR/PXY, which is expressed in procambium, perceives the TDIF signal [27, 30]. Defects in TDR result in an occasional loss of procambial cells between phloem and xylem. Immunolocalization studies of TDIF suggested that it is secreted from the phloem and perceived in procambial cells by TDR/PXY to regulate various aspects of procambium development [30]. Similar to the CLV3-WUS and CLE40-WOX5 pathways in SAM and RAM, *WOX4*, a member of the *WOX* gene family, is found to act downstream of the TDIF-TDR signaling pathway regulating procambial cell proliferation [28, 29]. A recent study demonstrated that GSK3 mediates TDIF-TDR signaling in the regulation of xylem differentiation. A transcription factor, BES1, a well-known target of GSK3s, acts downstream of TDIF-TDR-GSK3 pathway [33].

The high degree of sequence similarity of the cyst nematode CLE to TDIF led us to hypothesize that these nematodes secrete TDIF mimics to tap into the procambium developmental pathway to facilitate feeding site formation. In support of this hypothesis, we have shown that HsCLEB can function like TDIF in suppressing xylem differentiation and inducing the expression of genes related to procambial cell proliferation. The *tdr-1* mutant is insensitive to HsCLEB indicating the involvement of TDR for HsCLEB perception. Secondly, the expression of the TDIF-TDR-WOX4 pathway was examined during the course of nematode infection and was found to be expressed in feeding sites of parasitic J2 and J3 nematodes. TDR is expressed in the initial cell and cells surrounding the developing syncytium, consistent with a role in nematode CLE perception. In contrast, expression of *WUS* and *WOX5* were not detected in the syncytium, suggesting that WUS and WOX5 mediated stem cell maintenance is not needed for feeding site formation, although a target of CLV signaling must be required as syncytium formation is perturbed in *clv1-101 clv2-101 rpk2-5*. Third, nematode infection assays were performed on the corresponding TDIF-TDR-WOX4 pathway mutants. We observed a statistically significant reduction in nematode development on *cle41* and *tdr-1 wox4-1* mutants, but not *tdr-1* and *wox4-1* likely due to the limitations of the infection assay in picking up subtle developmental changes. Significantly, however, we showed a reduction in syncytium size with *cle41*, *tdr-1*, *wox4-1*, and *tdr-1 wox4-1* mutants, indicating the important role of the WOX4-mediated procambial cell proliferation pathway for successful feeding site formation. Furthermore, as the size of the feeding site is reduced in *cle41* mutants, both endogenous TDIF and nematode-derived B-type CLE peptides are required to generate a normal-sized syncytium.

The TDIF pathway is quite similar to the CLV3 and CLE40 pathways in that each is composed of a CLE peptide, LRR-RLKs, and a WOX homeodomain transcriptional regulator, and each plays a role in stem cell maintenance [8]. However, CLV3 and CLE40 suppress the expression of *WUS* and *WOX5*, respectively, which in turn restricts the stem cell population, whereas TDIF promotes the expression of *WOX4* to increase cell division in the stem cell population. One previous study revealed that A-type CLE peptides potentiate the action of B-type peptides. Type A and B peptides, in particular CLE6 and CLE41 peptides, act synergistically to induce cell proliferation within the vascular cambium and suppress differentiation [10]. This observation led us to propose that synergistic interaction may also occur during nematode feeding site formation especially considering that the nematode may be secreting both types of CLE peptides into the same feeding cell. To investigate this, we took advantage of a *clv1-101 clv2-101 rpk2-5* triple mutant, which blocks A-type CLE perception. Strikingly, we found that both *WOX4* and *CLE41* are downregulated in the *clv1-101 clv2-101 rpk2-5* triple mutant. This leads to a hypothesis whereby *WOX4* expression is reduced in *clv1-101 clv2-101 rpk2-5* at least partially through a reduction in signaling through TDR due to reductions in *CLE41* levels and suggests that A-type CLE peptides, in combination with CLE41, potentially regulate WOX4 activity via CLV1, CLV2 and RPK2 receptors. Exogenous treatment with both peptides from the nematode promotes massive cell proliferation, suggesting that nematode secreted A-type and B-type CLEs could potentially act synergistically to exert their function during feeding cell formation. While TDR was found to be a major player in the massive cell proliferation induced by simultaneous exogenous treatment of A- and B- type peptides, the underlying mechanism of this regulation needs further investigation.

An equally plausible scenario is that nematode A-type CLE peptides are required for infection, which in turn activates CLE41, and this is enhanced by nematode B-type CLE peptides for establishment of the feeding cell. During nematode infection, infective juvenile nematodes penetrate into the roots of host plants and migrate towards the vascular cylinder where they select a single cell to initiate a feeding site. In general, a pericycle or a procambial cell abutting xylem vessels is typically selected by cyst nematodes as the initial syncytial cell. Procambial cells and pericycle cells divide intensively and are incorporated into the syncytium or differentiate into a protective layer encircling the nematode feeding site [47, 48]. Thus, nematode CLEs may function as plant peptide mimics to hijack the TDIF-TDR-WOX4-mediated cell proliferation program already existing in the procambium. This is further supported by the decreased syncytium size observed in the receptor mutants. However, unlike the plant TDIF pathway, which is subject to spatial regulation, the nematode is capable of secreting B-type CLE peptides directly to the procambial cells. Secretion of CLE peptides by the nematode also has the potential to not only increase the local peptide concentration at the feeding site, but bypass the negative feedback regulation characteristic of endogenous CLE signaling pathways, and thus, constitutively activate pathways required for feeding cell maintenance. The level and ratio of A- and B-type peptide secreted by the nematode is also likely to be a critical factor for proper feeding cell establishment and is reflected in the significant expression differences found between A- and B-type peptides in the parasitic stages of the nematode.

In summary, this study has established a clear link between nematode CLE signaling and the WOX4-mediated cell proliferation pathway for feeding cell formation. Not only has this increased our understanding of molecular plant-nematode interactions, but has extended our knowledge of plant peptide signaling in plant developmental biology. Other than CLE peptides, recent evidence has suggested that cytokinin produced and secreted by the nematode is pivotal for the early activation of the cell cycle and cell division during expansion of feeding sites [49]. Given the increasing identification of nematode effectors and the complex developmental reprogramming required for feeding cell formation, a more complex picture is emerging that goes beyond a one effector-mediated signaling pathway for feeding site establishment. Investigations are intensively being pursued to discover potential interaction or crosstalk between plant and nematode CLE peptide and hormone signaling pathways.

## Materials and methods

### Plant material

The *clv1-101 clv2-101* double mutant [50] and the *rpk2-5* allele [36] have been described previously. The *clv1-101 clv2-101 rpk2-*5 triple mutant was generated by crossing *clv1-101 clv2-101* with *rpk2-5*. *cle41*, *tdr-1*, *wox4-1*, *tdr-1 wox4-1*, *pxy-3*, *pxy-3 wox4-1*, *pxy-3 wox14-1*, *pxy-3 wox4-1 wox14-1* have been described previously [27–29, 51]. TDRp:GUS, CLE41p:GUS and WOX4p:GUS lines were previously described and characterized [29, 30]. *clv1-101 clv2-101 tdr-1* triple mutant was generated by crossing *clv1-101 clv2-101* with *tdr-1*. WOX4p:GUS line was crossed into *tdr-1*, *cle41*, *clv1-101 clv2-101*, and *clv1-101 clv2-101 rpk2-5* mutants. CLE41p:GUS line was crossed into *clv1-101 clv2-101* and *clv1-101 clv2-101 rpk2-5* mutants. In order to generate TDRp:GFP construct, the promoter region was amplified from genomic DNA using primers (5’GGGGACAAGTTTGTACAAAAAAGCAGGCTCCCGGTTCTTCCACTACATCACGTAG’3 and 5’GGGGACCACTTTGTACAAGAAAGCTGGGTTCGTAGCTTTTAGAAAGAAATTAAAGTGAA’3), cloned into pDONRzeo vector, and subcloned into pGWB5 gateway vector. Then the construct was transformed into Col-0 with the floral dip method.

### Identification and cloning of B-type *CLE*s from cyst nematodes

The amino acid sequence of TDIF (HEVPSGPNPISN) from Arabidopsis was used to identify orthologous sequences in *H*. *glycines* by performing a TBLASTN search to a recently assembled transcriptome representing early parasitic life stages and filtering at an e-value cutoff of 1000. These candidate transcripts were then translated into protein and run through SignalP for prediction of N-terminal signal peptides, following which those candidates possessing a signal peptide were retained. Finally, the position of the CLE-domain identified was examined and any candidates lacking a C-terminal CLE domain were eliminated. Primers (HgCLEBF1: 5’ AGGAATAATTAACGGATTAAATCAA 3’ and HgCLEBR2: 5’ GAAGGAAAAGCATGAATAAACG 3’) were designed based on the UTR region sequences of SCN B-type *CLEs* to identify potential orthologous sequences from beet cyst nematode *H*. *schachtii*. Isolation of parasitic life stages, RNA extraction and first strand cDNA generation were conducted as previously described [14, 52]. PCR reactions were conducted with Q5 high fidelity Taq polymerase (New England Biolabs). PCR products were cloned into pGEM-T Easy vector (Promega) and sequenced. HgCLEB1, HsCLEB1 and HsCLEB2 cDNA sequences were deposited in Genbank under accession numbers KY271087, KY124382 and KY124383, respectively.

### Nematode infection on reporter lines

*H*. *schachtii* was propagated on sugar beet (*Beta vulgaris* cv. Monohi). Nematode eggs were isolated and hatched as previously described [36, 53]. After 2 days, J2 were collected and surface-sterilized. Arabidopsis seeds were sterilized and grown on modified Knop’s medium with Daishin agar (Brunschwig Chemie, Amsterdam, The Netherlands) [54]. Seven days after germination, seedlings were inoculated with 50 sterilized J2 per root.

### Histochemical promoter-GUS assays

At the indicated time points, seedlings were infiltrated with GUS staining solution (100 mM Tris, pH 7.0, 50 mM NaCl, 1 mM 5-bromo-4-chloro-3-indolyl-b-glucuronic acid, 1.0 mM potassium ferricyanide, pH 7.0, and 0.06% [v/v] Triton X-100) and incubated overnight at 37°C. The reaction was stopped with 70% ethanol. Stained roots were visualized with a Leica DM5500 microscope.

### Confocal microscopy

TDRp:GFP transgenic seeds were sterilized, grown and inoculated on glass slides. At the indicated time-points, infected roots were visualized with Leica TCS SP8 confocal microscope system excited at 488 nm with an emission filter of 500–550 nm.

### Nematode infection assay

Sterilized wild-type and mutant seeds (n = 36) were plated in 12-well Falcon tissue culture plates (BD Biosciences, San Jose, CA, USA) containing modified Knop’s medium with 0.8% Daishin agar in a randomized block design. Plants were grown at 24^°^C with a 12-h photoperiod. At 14 day post germination, seedlings were inoculated with 250 surface-sterilized J2. The J4 females were counted at 14 day post inoculation (dpi) and adult females were counted at 30 dpi. The average values were calculated and significant differences were determined by using Student’s *t*-test (*P* < 0.05).

To measure syncytium size, mutants were germinated on modified Knop’s medium in 6-well plates and inoculated at 14 days after germination with 200 surface-sterilized J2. At 14 dpi, syncytia that were transparent and fed upon by only one nematode were visualized with a Leica M205 stereo microscope. The syncytia were outlined using the AxioVision Release 4.8 (Carl Zeiss) and the area of the longitudinal section was calculated by the software. Significant differences were determined by using Student’s *t*-test (*P* < 0.05).

### Peptide treatment

For peptide treatment experiment with whole seedlings, seeds were germinated and grown in half-strength MS liquid medium for 7 d under continuous light with 120 rpm shaking. For cross-sectioning, seedlings were treated for 10 d under the same condition. For peptide treatment with root tissue, seedlings were grown on vertical plates for 6 d and transferred into liquid medium containing peptides and soaked for 24 h, then root tissue was collected for RNA extraction. HsCLE2 (RVSPGGPDPQHH), HsCLEB (HEVHypSGHypNPTQN), HsCLEB P9A (HEVHypSGHypNATQN), TDIF (HEVHypSGHypNPISN) and TDIF P9A (HEVHypSGHypNAISN) were synthesized with purity >95% (Genescript) and added to the medium at a final concentration of 1 or 5 uM.

### RNA extraction and quantitative PCR analysis

Nematode-infected root pieces were cut under a dissecting microscope at 5 dpi and kept in RNAlater RNA stabilization reagent according to the manufacturer’s instructions (Qiagen). For peptide treatment, whole roots or seedlings were collected and directly frozen in liquid nitrogen. Total RNA was extracted from root tissues using the RNeasy plant mini kit (Qiagen) and cDNA was synthesized using superscript III reverse transcriptase (Invitrogen) according to the manufacturer’s instructions. Quantitative PCR was performed using Applied Biosystem 7500 real-time PCR system. *AtUBC21* (At5g25760) was used as an endogenous control. Relative gene expression level was determined using ^ΔΔ^Ct method compared with the internal control. Primer sets used for qRT-pCR were described in S1 Table.

### Histological analysis

For cross sectioning, tissues were dissected and fixed with 2% glutaraldehyde in 50 mM PIPES buffer, pH 6.9 overnight. Samples were dehydrated in graded ethanol series and embedded in Technovit 7100 resin (Heraeus) according to the manufacturer's protocol. Sections of 2 uM were cut using an ultramicrotome (Reichert Ultracut S, Leica) and stained with 0.05% wt/vol toluidine blue O (Sigma-Aldrich) for 20 s. All samples were rinsed in tap water for 30 s to 1 min. After drying, the sections were mounted in DePex medium (EMS, Hatfield, PA) and covered with cover slips. Sections on slides were analyzed with a Leica DM5500 microscope.

### Xylem cell differentiation assay

Arabidopsis leaf-disc culture was performed as described previously [44]. Briefly, Arabidopsis seedlings were grown on half-strength MS agar plates for 3–4 weeks under continuous light. Leaf discs were carefully cut from rosette leaves 1 cm in length using a skin-biopsy punch 1 mm in diameter, then cultured in MS-based liquid culture (2.2 g/l MS salts, 50 g/l glucose containing 1.25 mg/l 2,4 –D, 0.25 mg/l kinetin, and 10 mM bikinin, pH5.7) in 12-well plates under continuous light with 120 rpm shaking. To dissect the effect of CLE peptide on xylem differentiation, leaf disc that were pre-cultured with bikinin for 24 h were washed twice with bikinin-free liquid medium, then cultured in medium with TDIF or HsCLEB for 48 h. The leaf disks were fixed in 1:3 mixture of acetic acid/ ethanol and mounted in Visikol clearing solution (Visikol Inc, New Brunswick, NJ). An Olympus IX70 was used to visualize the xylem cell differentiation.

## Supporting information

S1 Fig Isolation of B-type *CLE* genes from cyst nematodes. (A) Sequences of three transcripts (c21342_g1_i1; c21342_g1_i2, and c21342_g1_i5) identified from soybean cyst nematode aligned using ClustalW. TDIF sequence was used as a query to identify orthologous sequences from soybean cyst nematode transcriptome. The start and stop codons are highlighted in gray. The underlined UTR region sequences were used as primers to amplify homologous sequences from *H*. *glycines* and *H*. *schachitii* cDNA. (B) An alignment of the predicted protein sequences of the three *CLE* sequences from soybean cyst nematode using T-Coffee. The 12-aa CLE peptide sequence is highlighted in gray. Predicted signal peptide sequences are underlined. (C) Alignment of B-type CLE protein sequences from soybean cyst nematode and beet cyst nematode. CLE peptide sequence is highlighted in gray. Predicted signal peptide sequences are underlined. (D) Comparison of the relative expression levels of A- and B-type *CLEs* in the early parasitic *H*. *glycines* transcriptome in terms of TMM (trimmed mean of M-values)-normalized FPKM (fragments per kilobase of exon per million reads mapped). ppJ2, pre-parasitic second-stage juveniles; pJ2, parasitic second-stage juveniles.
(PDF)

S2 Fig **Arabidopsis WUSp:GUS (A) and WOX5p:GUS (B) expression analysis in nematode infection sites at 5 dpi. CLE41p:GUS (C-E), TDRp:GUS (F-H), WOX4p:GUS (I-K) expression in uninfected roots of Arabidopsis.** N = nematode; S = syncytium.
(PDF)

S3 Fig Nematode infection phenotype on Col-0, *pxy-3*, *pxy-3 wox4-1*, *pxy-3 wox14-1*, and *pxy-3 wox4-1 wox14-1*. Error bars represent SE of the means (n = 36). Asterisks indicate statistically significant differences compared with Col-0 by Student’s *t*-test (*P < 0.05 and **P < 0.01). The experiments were repeated three times with similar results.
(PDF)

S4 Fig Morphology and nematode infection phenotype of *clv1-101 clv2-101 rpk2-5* mutant. (A) Above-ground phenotype of 4-week-old wild type and *clv1-101 clv2-101 rpk2-5* mutant. (B) Wild type and *clv1-101 clv2-101 rpk2-5* seedlings grown on vertical plates. (C) Root length of wild type and *clv1-101 clv2-101 rpk2-5* mutant at 7 days post germination. Error bars represent SE of the means (n > 20). (D) Reduced nematode infection of *clv1-101 clv2-101 rpk2-5* mutant compared with wild type. Error bars represent SE of the means (n = 36). Asterisks indicate statistically significant differences compared with Col-0 by Student’s *t*-test (**P < 0.01).
(PDF)

S5 Fig Effect of exogenous treatment with TDIF, HsCLEB and HsCLE2 peptides on *WOX4* gene expression in roots. Seedlings including Col-0, *clv1-101 clv2-101 rpk2-5*, *tdr-1*, and *tdr-1 clv1-101 clv2-101*were grown on vertical plates for 6 d, then soaked in peptides for 24 h. (A) 5 μM TDIF P9A, 5 μM HsCLE2, and 5 μM TDIF were used. (B) 5 μM HsCLEB P9A, 5 μM HsCLE2, and 5 μM HsCLEB were used. Whole roots were cut for qRT-PCR to determine *WOX4* expression. Error bars represent SD of the means (n = 3). Asterisks indicate statistically significant differences by Student’s *t*-test (*P < 0.05 and **P < 0.01). Two biological replicates were performed.
(PDF)

S6 Fig Morphology and nematode infection phenotype of *tdr-1 clv1-101 clv2-101* mutant. (A)-(F) Cross-sections of wild type and *tdr-1 clv1-101 clv2-101* roots with and without nematode infection. (G) Diameter measurement of vascular sections in wild type and *tdr-1 clv1-101 clv2-101*. Error bars represent SE of the means (n > 7). (H) Reduced nematode infection of *tdr-1 clv1-101 clv2-101* and *clv1-101 clv2-101 rpk2-5* mutants. (I)-(K) Above-ground phenotype of *tdr-1 clv1-101 clv2-101* compared with *clv1-101 clv2-101* and wild type. Error bars represent SE of the means (n = 36). Asterisks indicate statistically significant differences compared with Col-0 by Student’s *t*-test (**P < 0.01). Three biological experiments were done.
(PDF)

S1 Table Primers used for qRT-PCR. (XLS)
