# Supplementary material for: Identification of cyst nematode B-type CLE peptides and modulation of the vascular stem cell pathway for feeding cell formation
Source: PLoS Pathog. 2017 Feb 3;13(2):e1006142. doi: 10.1371/journal.ppat.1006142 (PMC5319780; doi:10.1371/journal.ppat.1006142)
Supplement: S1 Fig — (A) Sequences of three transcripts (c21342_g1_i1; c21342_g1_i2, and c21342_g1_i5) identified from soybean cyst nematode aligned using ClustalW. TDIF sequence was used as a query to identify orthologous sequences from soybean cyst nematode transcriptome. The start and stop codons are highlighted in gray. The underlined UTR region sequences were used as primers to amplify homologous sequences from H. glycines and H. schachitii cDNA. (B) An alignment of the predicted protein sequences of the three CLE sequences from soybean cyst nematode using T-Coffee. The 12-aa CLE peptide sequence is highlighted in gray. Predicted signal peptide sequences are underlined. (C) Alignment of B-type CLE protein sequences from soybean cyst nematode and beet cyst nematode. CLE peptide sequence is highlighted in gray. Predicted signal peptide sequences are underlined. (D) Comparison of the relative expression levels of A- and B-type CLEs in the early parasitic H. glycines transcriptome in terms of TMM (trimmed mean of M-values)-normalized FPKM (fragments per kilobase of exon per million reads mapped). ppJ2, pre-parasitic second-stage juveniles; pJ2, parasitic second-stage juveniles. (PDF) [file ppat.1006142.s001.pdf]

A

```
c21342_g1_i1      CCTAATTAAATATAATAAAACAAATAAATATTTCCTGCAGGAATAATTAACGGATTAAAT
c21342_g1_i5      CCCAA------------------------GTTTG----AGGAATAATTAACGGATTAAAT
c21342_g1_i2      ------------------------------------------------------------

c21342_g1_i1      CAATTTGTCTTATAAATTGAAAGATAATCATGTCACAAATCAAAGCCTCGCTTCTTGTAG
c21342_g1_i5      CAATTTGTCTTATAAATTGAAAGATAATCATGTCACAAATCAAAGCCTCGCTTCTTGTAG
c21342_g1_i2      -------------------AAAGATAATCATGTCACAAATCAAAGCCTCGCTTCTTGTAG
                                     *****************************************

c21342_g1_i1      TGTTTTTGGCTTTTCTTATTATTAGCATCACTGAATCAAAGAAAAATGGCGATGATGATA
c21342_g1_i5      TGTTTTTGGCTTTTCTTATTATTAGCATCACTGAATCAAAGAAAAATGGCGATGATGATA
c21342_g1_i2      TGTTTTTGGCTTTTCTTATTATTAGCATCACTGAATCAAAGAAAAATGGCGATGATGATA
                  ************************************************************

c21342_g1_i1      ATCTGAATAATTCGAACGGAATGATGTCAAAGATTGGACGGATTTTCACTGGCGTATTGG
c21342_g1_i5      ATCTGAATAATTCGAACGGAATGATGTCAAAGATTGGACGGATTTTCACTGGCGTATTGG
c21342_g1_i2      ATCTGAATAATTCGAACGGAATGATGTCAAAGATTGGACGGATTTTCACTGGCGTATTGG
                  ************************************************************

c21342_g1_i1      CACTGTCATCCGTGGCACCGGGCGCGGCAAACGCTTCCCAAAAGGGTGGAGAAGTTCAAG
c21342_g1_i5      CACTGTCATCCGTGGCACCGGGCGCGGCAAACGCTTCCCAAAAGGGTGGAGAAGTTCAAG
c21342_g1_i2      CACTGTCATCCGTGGCACCGGGCGCGGCAAACGCTTCCCAAAAGGGTGGAGAAGTTCAAG
                  ************************************************************

c21342_g1_i1      CAGTTCAAAAACAACAATGTGCACCACCACCTCAAATGCCGGGCCGAGATTTG-------
c21342_g1_i5      CAGTTCAAAAACAACAATGTGCACCACCACCTCAAATGCCGGGCCGAGATTTG-------
c21342_g1_i2      CAGTTCAAAAACAACAATGTGCACCACCACCTCAAATGCCGGGCCGAGATTTGGTAAGAT
                  *****************************************************

c21342_g1_i1      ------------------------------------------------------------
c21342_g1_i5      ------------------------------------------------------------
c21342_g1_i2      TAGCAAGATGCATTAGTTATTCTTTAAAAGATTGGTCAATTAACGGGAAGAAGTTAAACA

c21342_g1_i1      --------------------------------------CATGAAGTTCCATCCGGGCCTA
c21342_g1_i5      --------------------------------------CATGAAGTTCCATCCGGGCCTA
c21342_g1_i2      AGAAAACTATTCTTATCATTAATTTTGTTTTTCAACAGCATGAAGTTCCATCCGGGCCTA
                                                        **********************

c21342_g1_i1      ATCCCACACAGAACAAAAAAGTTAACTTCTGAGGAAAAAAGAATATGCTTTCTTCGTTTA
c21342_g1_i5      ATCCCACACAGAACAAAAAAGTTAACTTCTGAGGAAAAAAGAATATGCTTTCTTCGTTTA
c21342_g1_i2      ATCCCACACAGAACAAAAAAGTTAACTTCTGAGGAAAAAAGAATATGCTTTCTTCGTTTA
                  ************************************************************

c21342_g1_i1      TTCATGCTTTTCCTTCTTTTTCTGATAACTTTTAGTAATTTATTCTTTGGGCATATTTAT
c21342_g1_i5      TTCATGCTTTTCCTTCTTTTTCTGATAACTTTTAGTAATTTATTCTTTGGGCATATTTAT
c21342_g1_i2      TTCATGCTTTTCCTTCTTTTTCTGATAACTTTTAGTAATTTATTCTTTGGGCATATTTAT
                  ************************************************************

c21342_g1_i1      CAATATATTTTAATCATTTTCCAC
c21342_g1_i5      CAATATATTTTAATCATTTTCCAC
c21342_g1_i2      CAATATATTTTAATCATTTTCCAC
                  ************************
```

B

```
c21342_g1_i1      MSQIKASLLVVFLAFLIISITESKKNGDDDNLNNSNGMMSKIGRIFTGVL
c21342_g1_i5      MSQIKASLLVVFLAFLIISITESKKNGDDDNLNNSNGMMSKIGRIFTGVL
c21342_g1_i2      MSQIKASLLVVFLAFLIISITESKKNGDDDNLNNSNGMMSKIGRIFTGVL
                  **************************************************

c21342_g1_i1      ALSSVAPGAANASQKGGEVQAVQKQQCAPPPQMPGRDL------------
c21342_g1_i5      ALSSVAPGAANASQKGGEVQAVQKQQCAPPPQMPGRDL------------
c21342_g1_i2      ALSSVAPGAANASQKGGEVQAVQKQQCAPPPQMPGRDLVRLARCISYSLK
                  **************************************

c21342_g1_i1      -----------------------HEVPSGPNPTQNKKVNF
c21342_g1_i5      -----------------------HEVPSGPNPTQNKKVNF
c21342_g1_i2      DWSINGKKLNKKTILIINFVFQQHEVPSGPNPTQNKKVNF
                                         *****************
```

Supplemental Figure 1

C

```
HgCLEB1         MSQIKASLLVVFLAFLIISITESKKNGD--DDNLNNSNGMMSKIGRIFTG
HsCLEB1         MPQIKASLLAVFLAFLIVSITESKKNADADADNGDNSTGIMSKIGRIFTG
HsCLEB2         MSQIKASLLVVFLGLIIISISESKKNAD--ADNGNNSTGMMS-IGRIFTG
                *.*******.***.::*:**:*****.*   ** :**.*:** *******

HgCLEB1         VLALSSVAPGAANASQKGGEVQAVQKQQCAPPPQMPGRDLHEVPSGPNPT
HsCLEB1         VLALSSVAPGAANASQTGGEVQAVQKQQWTPPPQMPGRDLHEVPSGPNPT
HsCLEB2         VLALSSVASGTANASQTGGEVQAVQKQQWTPPPQMPGRDLHEVPSGPNPT
                ********.*:*****.*********** :********************

HgCLEB1         QNKKVNF
HsCLEB1         QNKKVNF
HsCLEB2         QNKKVNF
                *******
```

D

	TMM-normalized FPKM expression	
**Transcript ID**	**ppJ2**	**pJ2**
**c29696_g3_i4 (*HgCLE2*)**	**0.66**	**1347.814**
**c21342_g1_i1**	**0**	**0.669**
**c21342_g1_i2**	**0.123**	**0.121**
**c21342_g1_i5**	**0.106**	**2.62**

Supplemental Figure 1
