# Supplementary figures and images for: Identification of cyst nematode B-type CLE peptides and modulation of the vascular stem cell pathway for feeding cell formation
Source: PLoS Pathog. 2017 Feb 3;13(2):e1006142. doi: 10.1371/journal.ppat.1006142 (PMC5319780; doi:10.1371/journal.ppat.1006142)

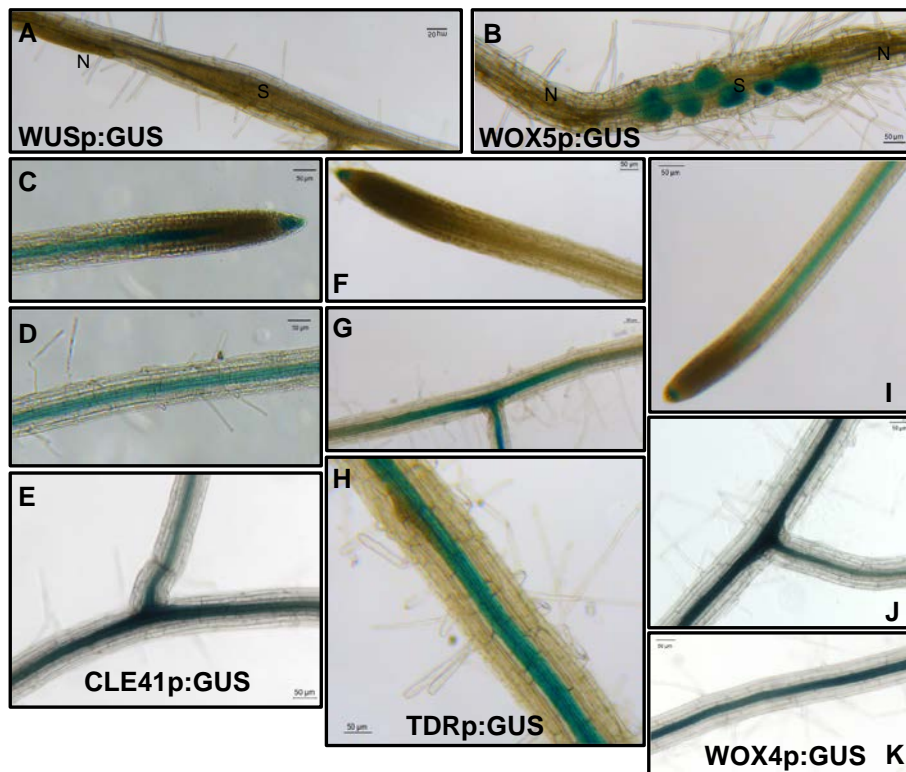

Supplemental Figure 2

Supplement: S2 Fig — Arabidopsis WUSp:GUS (A) and WOX5p:GUS (B) expression analysis in nematode infection sites at 5 dpi. CLE41p:GUS (C-E), TDRp:GUS (F-H), WOX4p:GUS (I-K) expression in uninfected roots of Arabidopsis. N = nematode; S = syncytium. (PDF) [file ppat.1006142.s002.pdf]

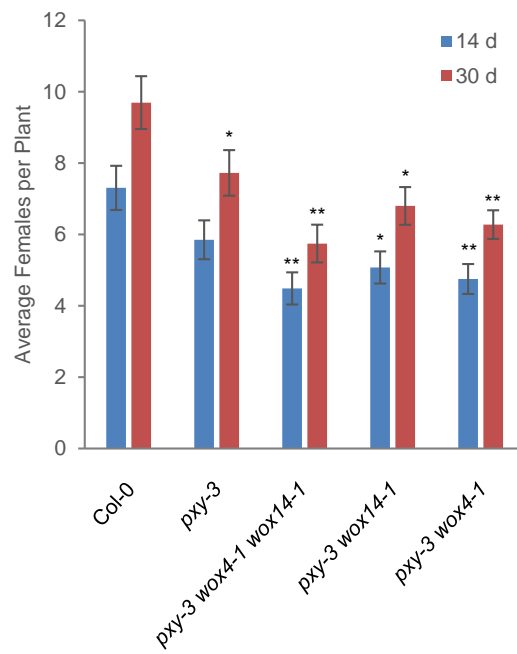

Supplemental Figure 3

Supplement: S3 Fig — Error bars represent SE of the means (n = 36). Asterisks indicate statistically significant differences compared with Col-0 by Student’s t-test (*P < 0.05 and **P < 0.01). The experiments were repeated three times with similar results. (PDF) [file ppat.1006142.s003.pdf]

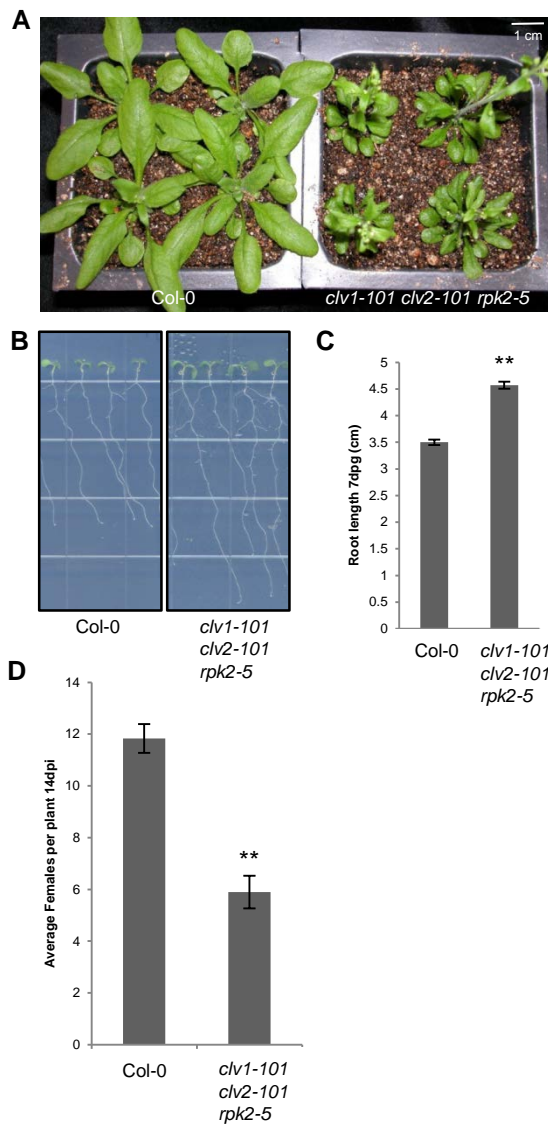

Supplemental Figure 4

Supplement: S4 Fig — (A) Above-ground phenotype of 4-week-old wild type and clv1-101 clv2-101 rpk2-5 mutant. (B) Wild type and clv1-101 clv2-101 rpk2-5 seedlings grown on vertical plates. (C) Root length of wild type and clv1-101 clv2-101 rpk2-5 mutant at 7 days post germination. Error bars represent SE of the means (n > 20). (D) Reduced nematode infection of clv1-101 clv2-101 rpk2-5 mutant compared with wild type. Error bars represent SE of the means (n = 36). Asterisks indicate statistically significant differences compared with Col-0 by Student’s t-test (**P < 0.01). (PDF) [file ppat.1006142.s004.pdf]

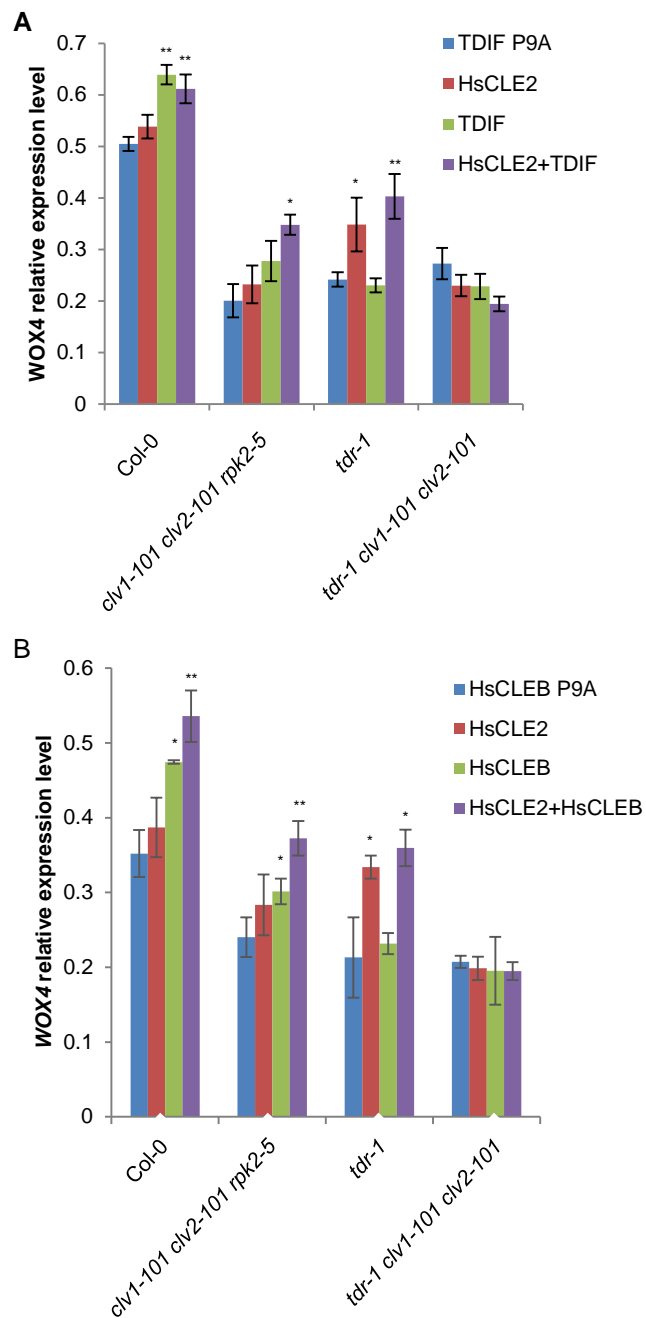

Supplemental Figure 5

Supplement: S5 Fig — Seedlings including Col-0, clv1-101 clv2-101 rpk2-5, tdr-1, and tdr-1 clv1-101 clv2-101were grown on vertical plates for 6 d, then soaked in peptides for 24 h. (A) 5 μM TDIF P9A, 5 μM HsCLE2, and 5 μM TDIF were used. (B) 5 μM HsCLEB P9A, 5 μM HsCLE2, and 5 μM HsCLEB were used. Whole roots were cut for qRT-PCR to determine WOX4 expression. Error bars represent SD of the means (n = 3). Asterisks indicate statistically significant differences by Student’s t-test (*P < 0.05 and **P < 0.01). Two biological replicates were performed. (PDF) [file ppat.1006142.s005.pdf]

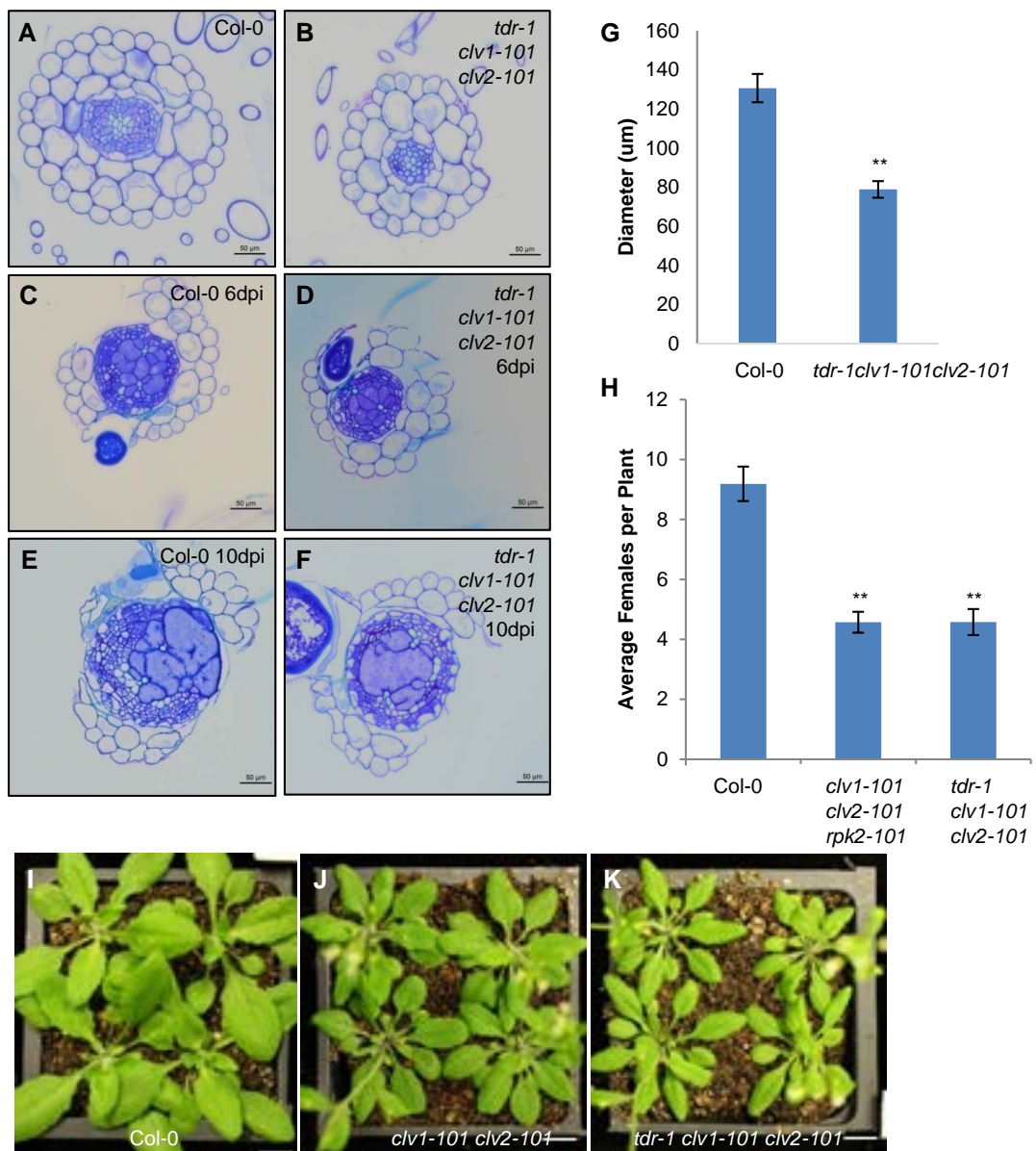

Supplemental Figure 6

Supplement: S6 Fig — (A)-(F) Cross-sections of wild type and tdr-1 clv1-101 clv2-101 roots with and without nematode infection. (G) Diameter measurement of vascular sections in wild type and tdr-1 clv1-101 clv2-101. Error bars represent SE of the means (n > 7). (H) Reduced nematode infection of tdr-1 clv1-101 clv2-101 and clv1-101 clv2-101 rpk2-5 mutants. (I)-(K) Above-ground phenotype of tdr-1 clv1-101 clv2-101 compared with clv1-101 clv2-101 and wild type. Error bars represent SE of the means (n = 36). Asterisks indicate statistically significant differences compared with Col-0 by Student’s t-test (**P < 0.01). Three biological experiments were done. (PDF) [file ppat.1006142.s006.pdf]
